# Alteration of structural and mechanical properties of the temporomandibular joint disc following elastase digestion

**DOI:** 10.1002/jbm.b.34660

**Published:** 2020-06-01

**Authors:** Sepanta Fazaeli, Fereshteh Mirahmadi, Vincent Everts, Theodoor H. Smit, Jan H. Koolstra, Samaneh Ghazanfari

**Affiliations:** ^1^ Department of Oral Cell Biology and Functional Anatomy, Academic Centre for Dentistry Amsterdam (ACTA) University of Amsterdam and Vrije Universiteit Amsterdam Amsterdam The Netherlands; ^2^ Department of Medical Biology Amsterdam University Medical Centers Amsterdam The Netherlands; ^3^ Aachen‐Maastricht Institute for Biobased Materials, Faculty of Science and Engineering Maastricht University Geleen The Netherlands; ^4^ Department of Biohybrid & Medical Textiles (Biotex) RWTH Aachen University Aachen Germany

**Keywords:** cartilage, elastin fiber, enzymatic degradation, mechanical properties, temporomandibular joint disc

## Abstract

The temporomandibular joint disc is a fibrocartilaginous structure, composed of collagen fibers, elastin fibers, and proteoglycans. Despite the crucial role of elastin fibers in load‐bearing properties of connective tissues, its contribution in temporomandibular joint disc biomechanics has been disregarded. This study attempts to characterize the structural–functional contribution of elastin in the temporomandibular joint disc. Using elastase, we selectively perturbed the elastin fiber network in porcine temporomandibular joint discs and investigated the structural, compositional, and mechanical regional changes through: (a) analysis of collagen and elastin fibers by immunolabeling and transmission electron microscopy; (b) quantitative analysis of collagen tortuosity, cell shape, and disc volume; (c) biochemical quantification of collagen, glycosaminoglycan and elastin content; and (d) cyclic compression test. Following elastase treatment, microscopic examination revealed fragmentation of elastin fibers across the temporomandibular joint disc, with a more pronounced effect in the intermediate regions. Also, biochemical analyses of the intermediate regions showed significant depletion of elastin (50%), and substantial decrease in collagen (20%) and glycosaminoglycan (49%) content, likely due to non‐specific activity of elastase. Degradation of elastin fibers affected the homeostatic configuration of the disc, reflected in its significant volume enlargement accompanied by remarkable reduction of collagen tortuosity and cell elongation. Mechanically, elastase treatment nearly doubled the maximal energy dissipation across the intermediate regions while the instantaneous modulus was not significantly affected. We conclude that elastin fibers contribute to the restoration and maintenance of the disc resting shape and actively interact with collagen fibers to provide mechanical resilience to the temporomandibular joint disc.

## 1 INTRODUCTION

The temporomandibular joint (TMJ) disc is a fibrocartilaginous tissue that overlies the articulating surfaces of the mandibular condyle and temporal fossa. The disc plays a crucial role in jaw kinematics (Tanaka & Koolstra, 2008; Tanaka & van Eijden, 2003). The extracellular matrix (ECM) of the disc is composed of collagenous network, elastin fibers, proteoglycans, and glycosaminoglycans (GAGs) (Detamore et al., 2005; Detamore & Athanasiou, 2003). The region‐dependent distribution and interaction of these elements yield to a heterogeneous and anisotropic mechanical behavior of the disc under various loading conditions during jaw movement (Tanaka et al., 2003).

The structural–functional characteristics of the disc can be compromised under pathological conditions (Farrar & McCarty, 1979; Schiffman et al., 2014). Although the etiology of disc pathology remains unknown, it has been suggested that the internal derangement may alter the ECM composition, subjecting it to abnormal loading conditions, which could lead to its degeneration (Tanaka, Detamore, & Mercuri, 2008). Structural–functional characterization of the individual structural elements of the TMJ disc can help to achieve a better understanding of the pathophysiology of the disc derangement, to establish design criteria for construction of a tissue‐engineered replica, and to develop more accurate computational prediction models.

Elastin fibers of different tissues have been characterized using selective enzymatic degradation techniques in combination with mechanical testing, biochemical analysis, or microscopic examination to assess the contribution of elastin fibers (Barbir, Michalek, Abbott, & Iatridis, 2010; Chow, Mondonedo, Johnson, & Zhang, 2013; Henninger, Underwood, Romney, Davis, & Weiss, 2013; Lee, Han, Lamm, Fierro, & Han, 2012; Lee, Midura, Hascall, & Vesely, 2001; Weisbecker, Viertler, Pierce, & Holzapfel, 2013; Yapp & Chen, 2015). In response to loading, connective tissues exhibit heterogeneous, anisotropic, and hyperelastic behavior where expansion of the tissue is restricted by collagen (Broom & Marra, 1985; Thambyah & Broom, 2006) or elastin fibers (Roach & Burton, 1975). Collagen fibers are responsible for providing stiffness and strength to the tissue at higher strains while elastin fibers accounts for its elasticity (Roach & Burton, 1975; Schriefl, Schmidt, Balzani, Sommer, & Holzapfel, 2015). Elastin fibers store elastic energy, thereby preserving collagen from damage through impact loading (Henninger et al., 2013; Lee et al., 2001; Reihsner, Menzel, Mallinger, & Millesi, 1991; Smith, Byers, Costi, & Fazzalari, 2008; Yapp & Chen, 2015), which is particularly relevant for compliant structures like the TMJ disc.

While the mechanical contribution of collagen fibers has been extensively studied, there are only few studies on the occurrence and distribution of elastin fibers in the disc (Clément et al., 2006; Detamore et al., 2005; Gross, Bumann, & Hoffmeister, 1999; Iwai‐Liao, Ogita, Tsubai, & Higashi, 1994; Keith, 1979; O'Dell, Burlison, Starcher, & Pennington, 1996). Therefore, the aim of the present study is to provide a structural–functional characterization of elastin fibers in the TMJ disc, in light of their contribution in healthy and degenerated conditions. We used enzymatic degradation of elastin fibers in combination with microscopic, biochemical and mechanical analyses to elucidate the extent of elastin fiber contribution to TMJ disc biomechanics. We investigated five different regions known to possess a different quantity and organization of ECM constituents (Scapino, Obrez, & Greising, 2006): posterior band (PB), anterior band (AB), intermediate zone central (IZC), intermediate zone medial (IZM), and intermediate zone lateral (IZL).

## 2 MATERIALS AND METHODS

### 2.1 Sample preparation

Ten young porcine heads were obtained from a local abattoir immediately after slaughter. The TMJ discs with intact condylar heads were harvested en bloc and were removed from extraneous parts. The average size of the TMJ discs was approximately 3 mm × 1.5 mm with a thickness varying between 1.5 and 4 mm. Next, the discs were washed in phosphate buffered saline (PBS), wrapped in gauze soaked in PBS and protease inhibitors (Roche Diagnostics, Germany), and stored at −20°C until use.

### 2.2 Enzymatic digestion

Based on previous studies (Barbir et al., 2010; Lee et al., 2001; Schriefl et al., 2015; Smith et al., 2008), different concentrations of elastase (1 U/ml, 3 U/ml, and 5 U/ml) were used to degrade elastin fibers in different tissues while samples maintained sufficient integrity. We used pancreatic elastase (Sigma, MO), as a potent elastin degrading enzyme, in combination with soybean trypsin inhibitor (SBTI) (Sigma, MO) to minimize nonspecific enzymatic activity of elastase (Kafienah, Buttle, Burnett, & Hollander, 1998). Preliminary tests revealed that 16 hr of treatment in a physiological solution at 37°C with a volume activity of 3 U/ml elastase and 0.1 mg/ml SBTI was sufficient for the purpose of this study.

The left disc of each head was assigned to the control (PBS) group (*n* = 5) and the right disc to the treated (elastase) group (*n* = 5). All discs were mechanically tested before and after incubation with PBS or elastase, thus serving as their own controls. Samples were distributed to vials containing 12 ml PBS or elastase solution and incubated accordingly. Following treatment, the samples were washed in three changes of PBS for 5 min each before undergoing the post‐treatment mechanical test.

### 2.3 Immunofluorescence

To visualize the effect of elastase treatment, three pairs of disc were used for immunohistochemistry and included in the PBS or elastase treatment. Porcine arteries were used as positive controls for elastin staining. Following treatment, samples were washed with PBS, fixed overnight in 4% paraformaldehyde, and the central, lateral and medial parts of the disc were cut into two parts, one used for transverse and the other for sagittal sectioning. Then, each part was sectioned at 10 μm.

The sections were incubated with PBS followed by blocking buffer (1% BSA, 20% goat serum, PBS) for 20 min and incubated with a cocktail of primary antibodies containing rabbit polyclonal anti‐collagen I (1:1000, ab34710, Abcam, MA) and mouse polyclonal anti‐elastin (1:100, ab9519, Abcam, MA) overnight at 4°C. Thereafter, sections were washed with PBS, and a cocktail of secondary antibodies containing Alexa 488 goat anti‐rabbit (1:2000; Invitrogen, CA) and Alexa 647 goat anti‐mouse (1:200; Invitrogen, CA) was added for 1 hr. Then, sections were washed with PBS, stained with DAPI for 10 min, washed in PBS and mounted with Vectashield mounting medium (Vector laboratories, CA). Images were obtained with an inverted confocal microscope (Leica SP8, Germany).

### 2.4 Transmission electron microscopy (TEM)

Cartilage samples were fixed at ambient temperature in 4% paraformaldehyde and 1% glutardialdehyde in 0.1 M sodium cacodylate buffer for 24 hr. The samples were subsequently washed in buffer and postfixed in 1% OsO4 for 1 hr. Then, the tissue was dehydrated in a series of ethanol and embedded in epoxy resin. Ultrathin sections were made using a diamond knife. Sections were stained with uranyl acetate and lead nitrate and examined in a CM10 Philips electron microscope.

### 2.5 Collagen tortuosity analysis

Collagen tortuosity index, as a measure of waviness, was calculated from the microscopy images based on the Gabor wavelet algorithm developed in MATLAB (Mathwork, MA) (Ghazanfari et al., 2015). In short, Gabor wavelets with a range of different wavelengths (2, 3, 4, and 5) and orientations (0, π/4, π/2, and π) were convolved with the images, and the corresponding histograms were obtained. The tortuosity index was then calculated by deducting the maximum number in the Gabor histograms from 1. Thus, the tortuosity index values varied between 0 and 1. If the tortuosity index is 0, the fibers are completely straight, and if tortuosity index is 1, the tortuosity of fibers is maximum.

### 2.6 Cell shape analysis

Using DAPI images, cell shape index was quantified in MATLAB (Mathwork, MA) as previously described (Ghazanfari et al., 2010; Haghighipour, Tafazzoli‐Shadpour, Shokrgozar, & Amini, 2010; van Esterik et al., 2017). In brief, a binary threshold was applied to the gradient images after the images were converted to a grey‐scale format. After removal of small artifacts by a geometrical filter (Figure S1), Shape Index (SI) as a measure of cell elongation was calculated as SI = (4π × area)/perimeter^2^. If a cell is fully circular, it has a shape index of 1, and if it is a line, it has a shape index of zero.

### 2.7 Tissue volume quantification

The volume of the samples before and after PBS/elastase treatment was measured using a measuring cylinder filled with 20 ml of PBS. The disc was placed inside the cylinder causing an increase of PBS level, which was marked and imaged accordingly. Then, the difference between the marked points using the ImageJ (NIH, MD) was measured to calculate the volume changes.

### 2.8 Biochemical analysis

Two adjacent specimens from each of the five mechanically tested regions were excised using a 4‐mm dermal punch. From each pair of adjacent specimens, one was assigned to hydroxyproline and GAG content measurement and one for elastin quantification. The total hydroxyproline and GAG content were determined according to Ghazanfari et al. (Ghazanfari et al., 2015). Accordingly, the assigned specimen was lyophilized overnight, dry weighted and digested by papain (Sigma, MO) for 16 hr. GAG content was quantified by 1‐9‐dimethylmethylene blue dye‐binding assay using chondroitin sulfate from shark cartilage (Sigma, MO) to create a standard curve. The hydroxyproline, as a measure of collagen content, was determined by alkaline autoclaving of the papain digest followed by spectrophotometric quantification of its reaction with chloramine‐T and dimethylaminobenzaldehyde. The elastin content was quantified following the Fastin elastin colorimetric assay protocol (Biocolor Ltd., UK). As previously described (Ghazanfari, Driessen‐Mol, Hoerstrup, Baaijens, & Bouten, 2016), samples were wet weighted and digested by oxalic acid, solubilizing insoluble elastin into α‐elastin polypeptides, which later were quantified along with soluble tropoelastin spectrophotometrically.

### 2.9 Experimental apparatus

To characterize the mechanical properties of the samples, we used a custom‐built material testing machine (Berendsen et al., 2009) with a resolution of 1 μm at a rate of maximally 30 Hz. It consists of two 4 mm circular flat‐ended indenters and a chamber. One indenter is fixed at the center inside the chamber and aligned with the upper indenter, which applies cyclic compressive displacement controlled by a custom‐made software (implemented in LabVIEW 8.2, TX). A 25 N load cell was used to register the normal reaction force applied to the top indenter and was connected to a bridge amplifier. After A/D conversion, the signals were registered by the same software controlling the displacing indenter. Displacement and reaction force were recorded simultaneously at a 16 ms interval.

### 2.10 Mechanical loading experiment

We determined the regional mechanical properties of the samples using the protocol developed previously (Fazaeli, Ghazanfari, Everts, Smit, & Koolstra, 2016). Using cyanoacrylate, the inferior surface of the desired region of the disc was glued to the bottom indenter, and the chamber was filled with PBS. Next, a tare load of 0.02 N was applied. Following 5 min of relaxation, the platen‐to‐platen distance was measured by a digital caliper as the sample thickness. To obtain a reproducible loading pattern, samples were preconditioned for 3 min through a series of cyclic compressive displacements corresponding to 10% strain at a frequency of 1 Hz. Following 5 min recovery time, samples were loaded for another 20 cycles similar to the preconditioning loading protocol. When done, the disc was carefully detached and glued at another region and mechanically tested. The loading protocol was chosen based on the physiological loading during human mastication (Druzinsky, 1993).

Stress and strain were driven from load–displacement data using routines written in Matlab (MathWorks, MA). For evaluation of the mechanical data, we focused on the following terms: (a) instantaneous modulus defined as the ratio of maximum stress occurring at the first cycle to its corresponding strain and; (b) maximum hysteresis calculated from the enclosed area of the first loop of stress–strain curve, called maximum energy dissipation.

### 2.11 Statistics

Two‐way ANOVA with repeated measurements was applied to compare two overall factors of elastase treatment and disc region on the mechanical and biochemical measurements of the associated groups. A Bonferroni post hoc test was performed to investigate the regional differences within each group. Additionally, a paired Student's *t* test determined the differences between the corresponding single regions of the control and treated groups.

To analyze the differences of collagen tortuosity and cell shape indices between the control and treated groups, we calculated the average of indices in separate regions and then compared the mean of all regions indices between the two groups using an unpaired Student's *t* test. Furthermore, a paired Student's *t* test was used to compare the differences between the average of disc volumes in the control and elastase treated groups. Statistical analyses were performed using GraphPad Prism 6.01 (GraphPad, CA) using a significance level of *p* < .05.

## 3 RESULTS

### 3.1 Immunofluorescence

**FIGURE 1:**
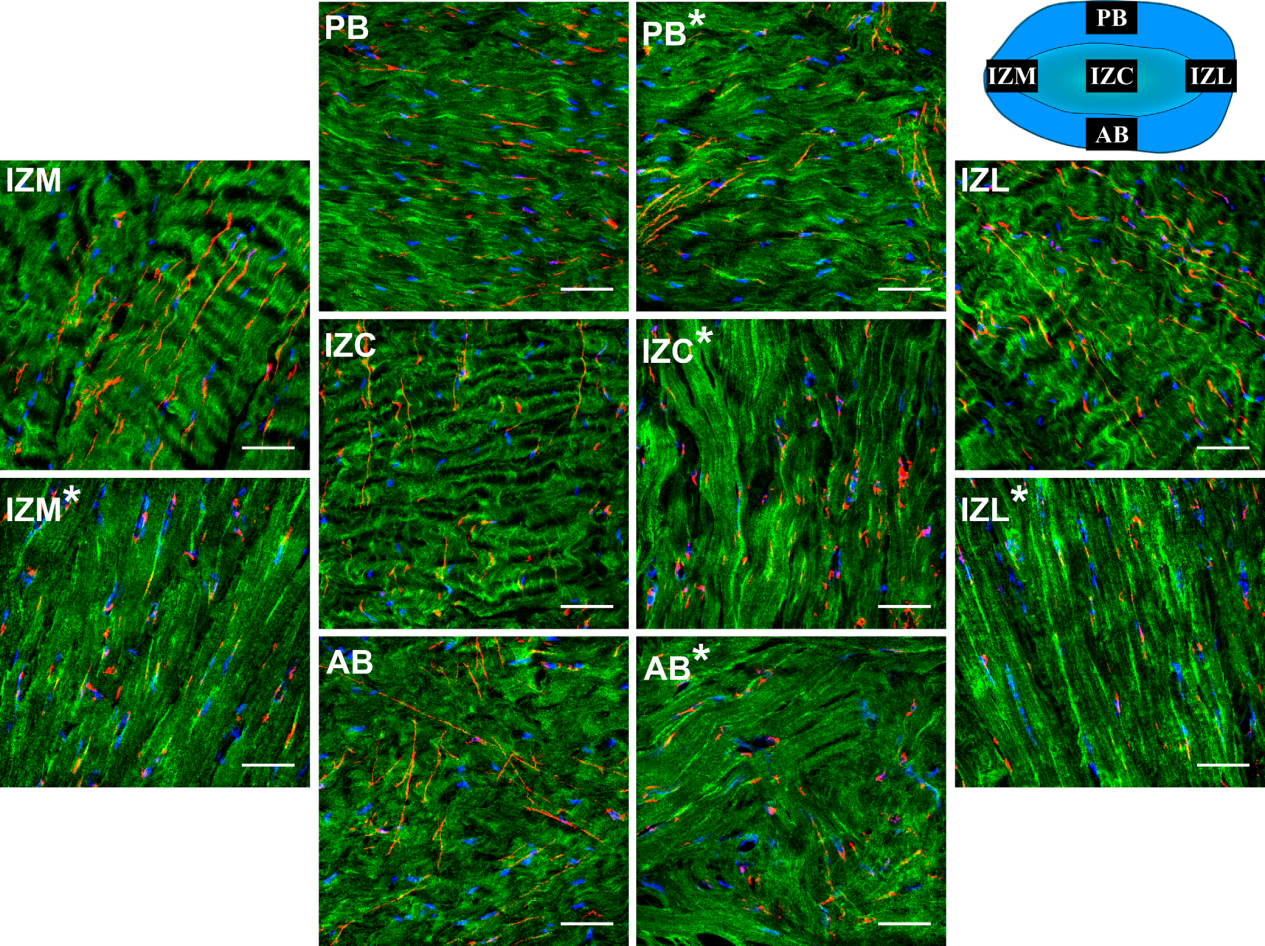
Top view confocal image and overlay immunofluorescence staining of elastin fibers, collagen fibers type I and cell nuclei of porcine TMJ disc before and after elastase treatment. Elastin fibers, collagen fibers type I and cell nuclei can be distinguished in red, green, and blue, respectively. The schematic configuration of the TMJ disc (seen from top), placed in the uppermost right corner of the figure exhibits the location of different regions of the TMJ disc and the direction of imaging (top view). Region labels with asterisk present the treated (elastase) samples. Anteroposteriorly aligned collagen fibers in the intermediate regions (IZC, IZM, and IZL) merge with fibers at the peripheral bands (PB and AB), forming a dense collagenous network. Note the frequent oblique elastin fibers criss‐crossing through the isotropic collagenous network at the AB, while in other regions elastin fibers are mainly aligned parallel with collagen fibers. Following the elastase treatment (regions with asterisk) the elastin fibers are fragmented, patched and diminished across the TMJ disc. Also, note the reduction of collagen fibers tortuosity and cell nuclei elongation following the elastase treatment (regions with asterisk), with more noticeable impact in the intermediate regions (IZC, IZM, and IZL). Scale bar: 50 μm. For more clarity, refer to Figures [Link], [Link], presenting immunofluorescence staining of elastin fibers, collagen fibers type I and cell nuclei in separate channels, respectively

**FIGURE 2:**
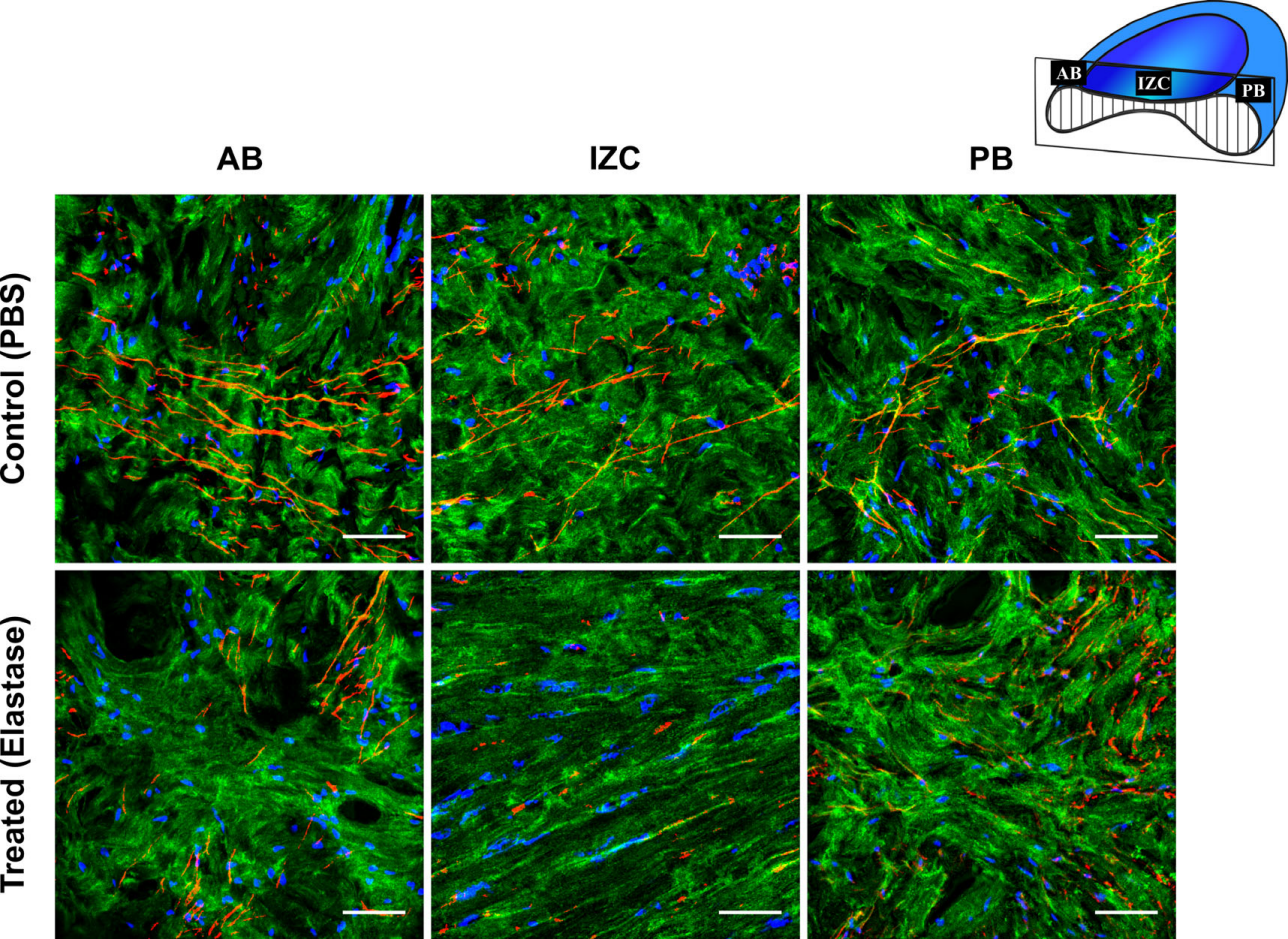
Sagittal view confocal image and immunofluorescence staining of elastin fibers, collagen fibers type I and cell nuclei of porcine TMJ disc before and after elastase treatment. Elastin fibers, collagen fibers type I and cell nuclei can be distinguished in red, green, and blue, respectively. The schematic configuration of the TMJ disc sagittal cross‐section, placed in the uppermost right corner of the figure, exhibits the location of different regions of the TMJ disc. The upper row shows images of different regions in the control (PBS) samples and the lower row shows the treated (elastase) ones. The collagen fibers, running anteroposteriorly in the IZC, extend to the AB and PB where they merged with fibers mainly aligned mediolaterally (perpendicular to the plane). The elastin fibers, running parallel to the collagen fibers in the IZC, also extend to the AB and PB where they show less directivity. Following the elastase treatment, the elastin fibers are fragmented, patched and diminished across the TMJ disc sagittal cross‐section. Also, note the reduction of collagen fibers tortuosity and cell nuclei elongation following the elastase treatment with more noticeable impact in the intermediate regions (IZC, IZM, and IZL). Scale bar: 50 μm. For more clarity, refer to Figures [Link], [Link], presenting immunofluorescence staining of elastin fibers, collagen fibers type I and cell nuclei in separate channels, respectively

The immunofluorescence staining of nontreated control samples revealed straight, long and branching elastin fibers interlacing heterogeneously through highly organized collagenous network across the disc (Figures 1 and 2). Collagen fibers were anteroposteriorly oriented in the intermediate regions, merging to encircling fibers at the periphery (Figures 1 and S4). The elastin fibers were occasionally branching at acute angles and reuniting into straight or oblique fibers with a preferential alignment parallel to collagen fibers (Figures 1 and S3). In the AB where the collagen fibers seemed more isotropic, elastin fibers appeared more oblique with higher branching frequency forming random patterns (Figures 1 and S3). Sagittal sections further demonstrated the presence of elastin fibers throughout the full thickness of the disc (Figure 2, Figure S2 and S6) with straight elastin fibers in the IZC running along the collagen fibers and extending to the AB and PB where they display more branching and random patterns of distribution.

Immunofluorescence staining of the treated samples clearly showed loss of elastin compared to the control group (Figure 2). The effect of degradation was most pronounced in the thinnest zone of the disc, intermediate region (IZC, IZM, and IZL), leading to extensive diminishing of elastin fibers and sporadic remnants of elastin fragments (Figures 1 and 2, Figures S3 and S6). In the bands (AB and PB) however, the elastase incubation resulted in less fragmentation but enough to create regions containing loose and coiled elastin fibers (Figures 1 and 2, Figures S3 and S6). The orientation of collagen fibers seemed unaffected by elastase degradation (Figures 1 and 2, Figures S4 and S7). Following elastase treatment, we observed severe degradation of elastin fibers compared to the control sample (Figure S9).

### 3.2 Tissue volume

**FIGURE 3:**
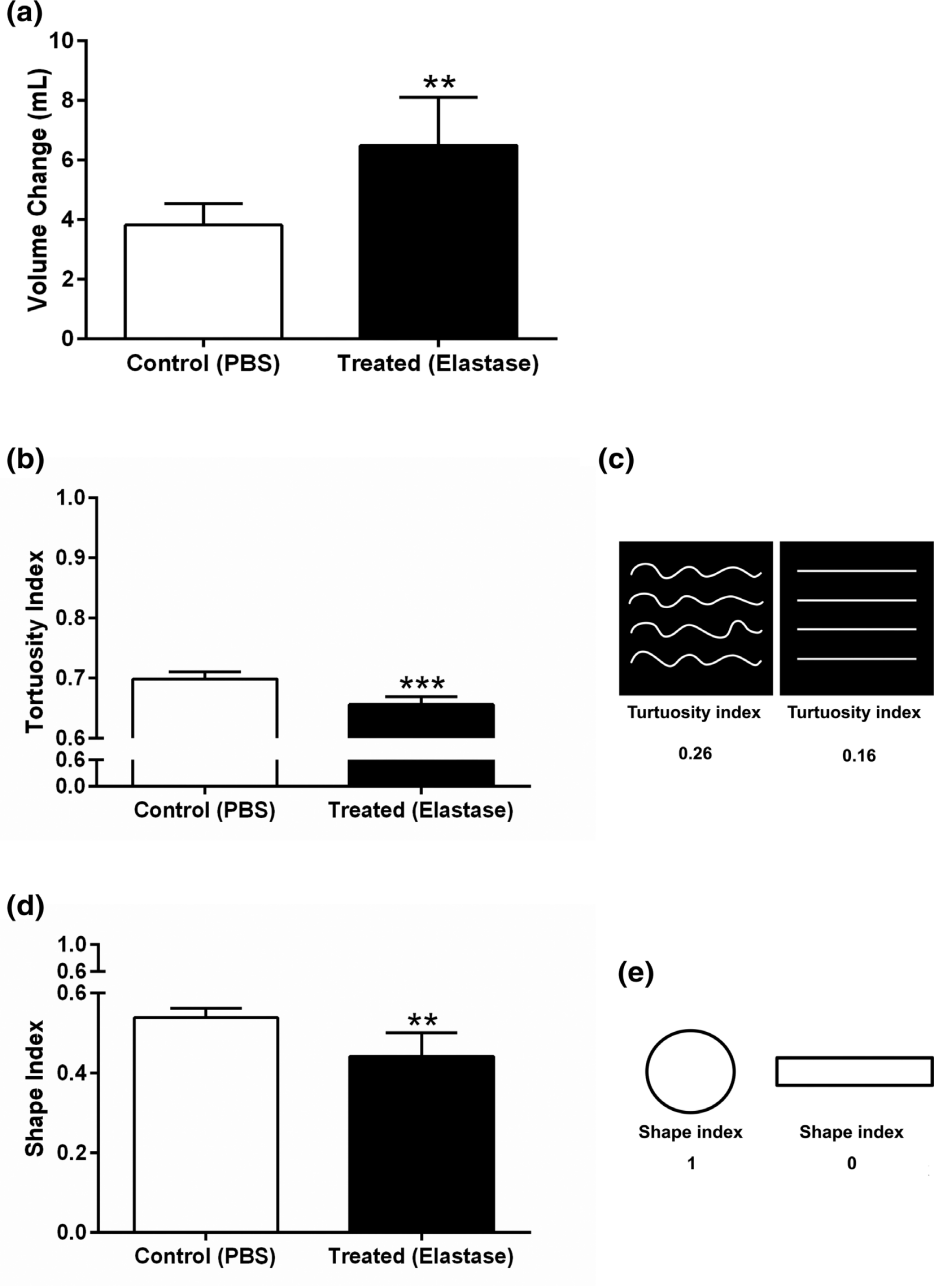
Effect of elastase treatment on volume, cell shape, and collagen fibers tortuosity of the porcine TMJ disc. (a) TMJ disc volume. (b) Average of quantified tortuosity indices obtained from all regions of the TMJ disc. (c) Average of quantified cell shape indices obtained from all regions of the TMJ disc. All data are presented as mean ± *SD*. ** indicates *p* < .01, and *** indicates *p* < .001

The disc volume before and after treatment showed a significant increase in the treated group (Figure 3a).

### 3.3 Collagen tortuosity

The degree of fiber tortuosity in the treated samples was significantly smaller than the control samples (Figure 3b). As the fibers' tortuosity decreased from left to right, their corresponding indices decreased accordingly (Figure 3c).

### 3.4 Cell shape

The results presented in Figure 3d show that the cells were significantly elongated following elastase treatment. The shape index changed from 1 to 0 as the shape geometry shifted from line to circle (Figure 3e).

### 3.5 Biochemical analysis

**FIGURE 4:**
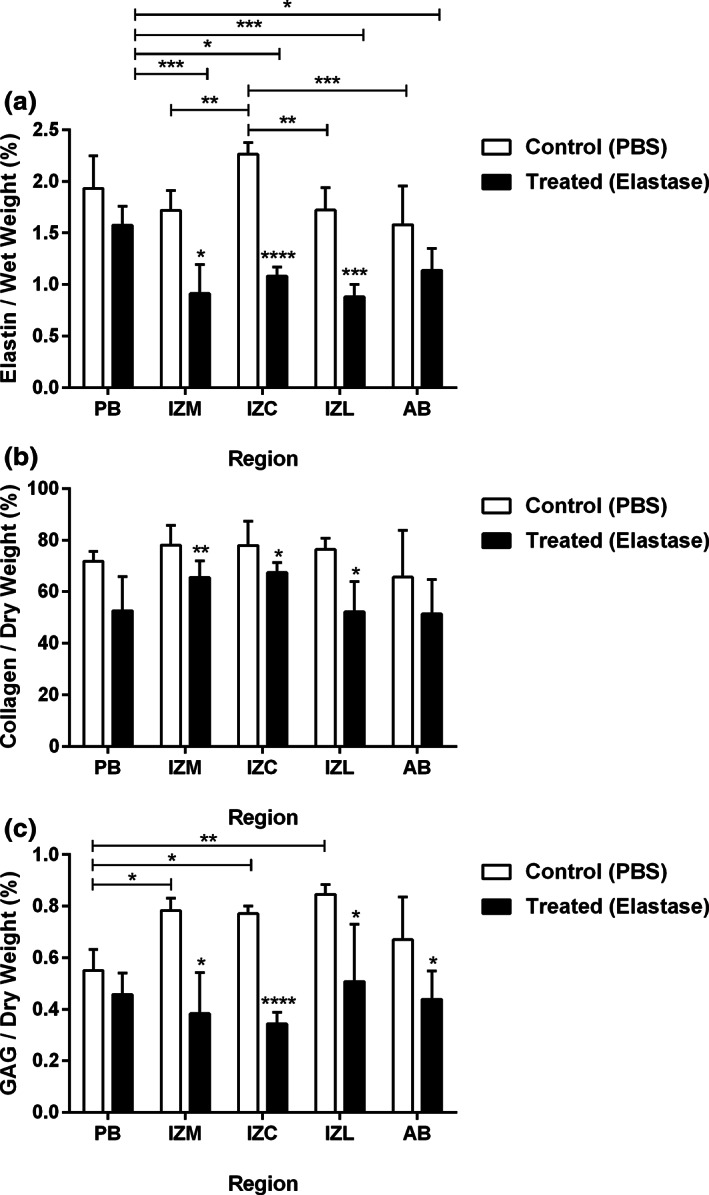
Effect of elastase treatment on elastin (a), collagen (b), and GAG (c) content of the porcine TMJ disc. After elastase treatment, the elastin content was significantly reduced in the intermediate regions (IZC, IZM, and IZL) and the heterogeneous distribution of elastin content was changed. Elastase treatment resulted in a significant reduction of collagen content in the intermediate regions (IZC, IZM, and IZL). The elastase treatment significantly reduced the GAG content in the intermediate regions (IZC, IZM, and IZL) and AB. All data are presented as mean ± *SD*. * indicates *p* < .05, ** indicates *p* < .01, *** indicates *p* < .001 and **** indicates *p* < .0001

The elastin content of the control and treated groups are presented in Figure 4a. Elastase treatment reduced the average elastin content of the disc by 40%. Two‐way ANOVA showed an overall significant effect of elastase treatment (*p* < .0001) and disc region (*p* < .0001) on the elastin content across the disc. Elastase treatment significantly reduced elastin content across the thinnest regions as in IZC (52%), IZM (47%), and IZL (49%). Post hoc testing showed regional differences of elastin content in the control group with IZC possessing the highest amount of elastin, which was significantly different from IZM, IZL and AB. In the treated samples, however, regional variation of the elastin content have a different pattern with PB containing the highest remnant of elastin, which was significantly different from IZC, IZM, IZL, and AB.

The collagen content of the control and treated groups are presented in Figure 4b. Following elastase treatment, the average collagen content of the disc was reduced by 22%. Two‐way ANOVA revealed an overall significant effect of elastase treatment (*p* < .0001) and disc region (*p* < .05) on collagen content across the disc. Additionally, analysis of the collagen content between the control and treated groups within a single region showed significant reduction of the collagen content across the thinnest regions as in IZC (13%), IZM (16%), and IZL (32%).

Figure 4c shows the GAG content of the control and treated samples. Following elastase treatment, there was an average of 41% reduction in GAG content of the disc. Two‐way ANOVA revealed an overall significant effect of elastase treatment (*p* < .0001) and disc region (*p* < .05) on the GAG content. Specifically, the effect of elastase treatment was significantly pronounced in the IZC (55%), IZM (51%), IZL (40%), and AB (35%). Furthermore, post hoc test expressed regional variation of the GAG content in the control group as PB possesses significantly lower amount of GAG compared to IZC, IZM, and IZL. The regional heterogeneity of GAG content was lost following the treatment.

### 3.6 Mechanical properties

**FIGURE 5:**
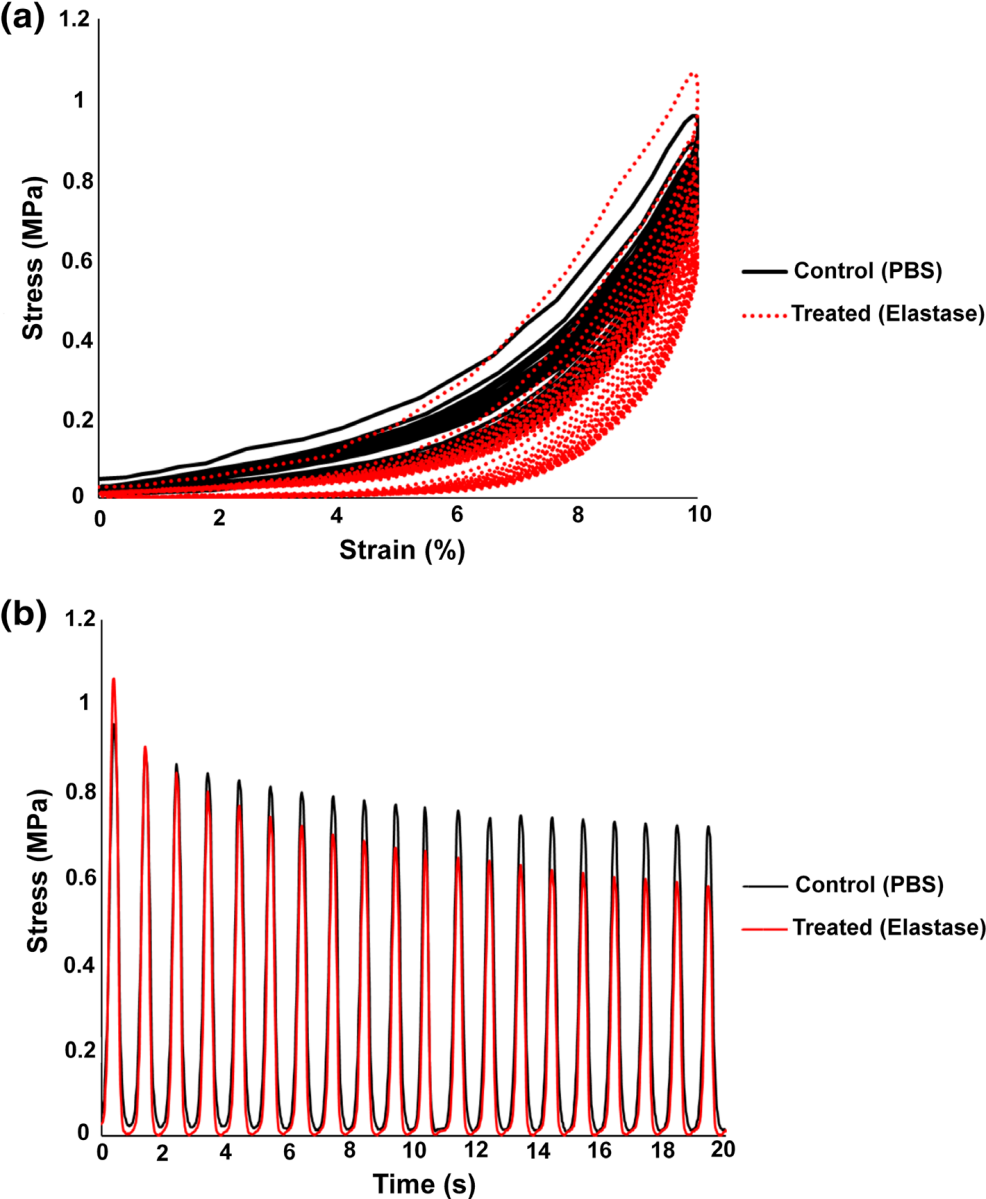
Effect of elastase treatment on the stress response of the intermediate regions (IZC, IZM, and IZL) in the porcine TMJ disc during cyclic loading. The control (PBS) data (black) and treated (elastase) data (red) indicate the mechanical changes induced by elastase treatment in (a) mechanical hysteresis graph and (b) stress‐time graph. Note the increase of maximal energy dissipation (a) and the continuous softening behavior of the treated (elastase) disc (a and b)

Figure 5 shows the representative mechanical behavior of the IZC in the disc before and after elastase treatment. The stress–strain curve (Figure 5a) showed a nonlinear behavior and a Mullins effect also after the elastase treatment. The average of maximal energy dissipation across the disc was increased by approximately 40% following the elastase treatment and the stress–strain curve continuously shifted down while that of the control relaxed after a few consecutive cycles (Figure 5a). The continuous downward shifting of the stress–strain curve is partially reflected in the corresponding stress–time graph (Figure 5b) as the stress peaks did not level off like the control disc.

**FIGURE 6:**
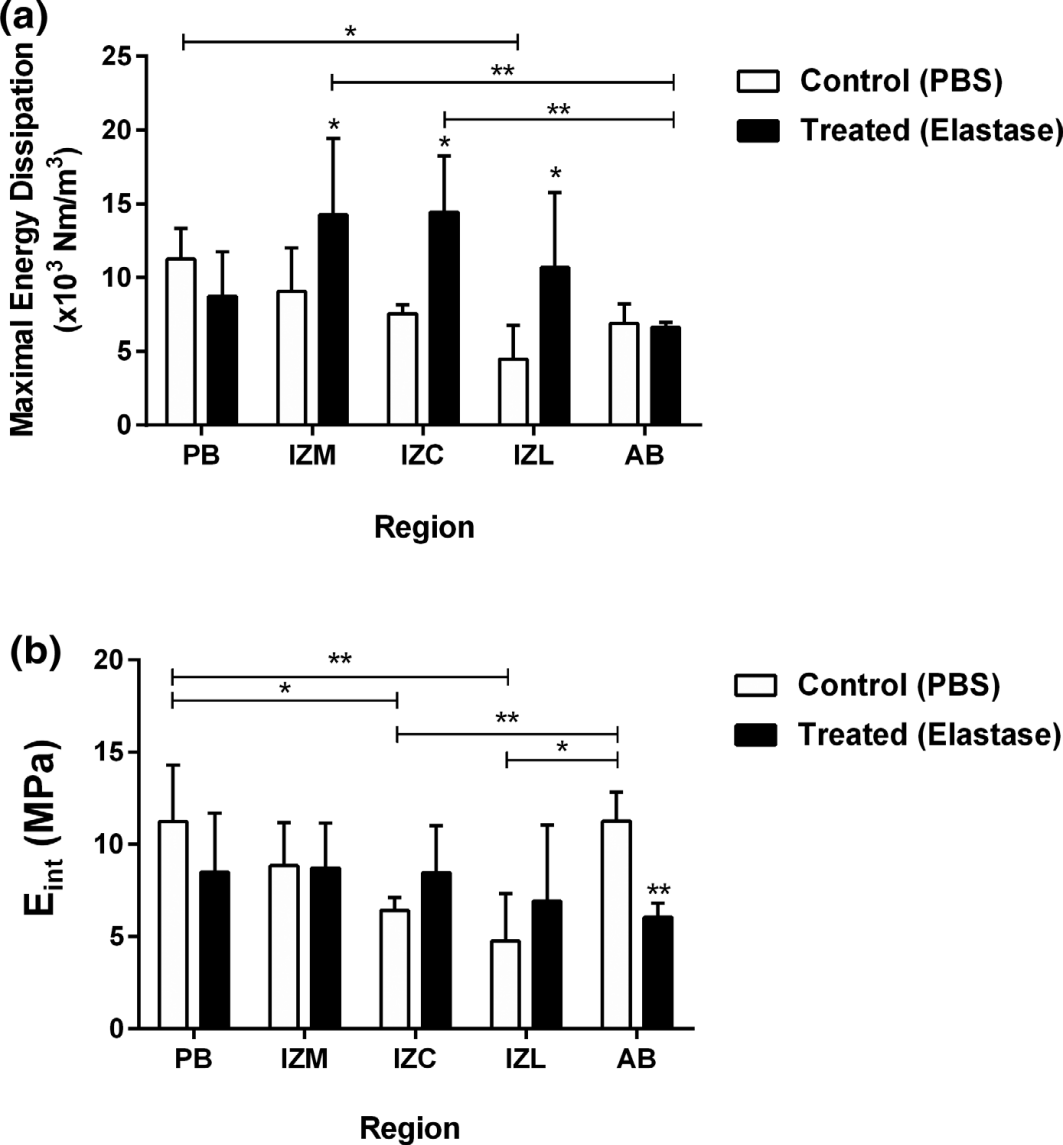
Effect of elastase treatment on mechanical compressive properties of the porcine TMJ disc. (a) Following elastase treatment, the maximal energy dissipation was significantly increased in the intermediate regions (IZC, IZM, and IZL) while decreased minimally in the AB and PB. The regional variation in the control (PBS) samples was only observed between PB and IZL, with the former being significantly higher. However, this pattern was changed after elastase treatment with IZC an IZM being higher than AB. (b) The effect of elastase treatment on the instantaneous modulus of the disc found to be pronounced only in the AB where the stiffness reduced significantly. Also, there seemed to be a general increasing trend in the instantaneous modulus of the intermediate regions. All data are presented as mean ± *SD*. * indicates *p* < .05, and ** indicates *p* < .01

Two‐way ANOVA revealed a significant effect of elastase treatment (*p* < .001) and disc region (*p* < .05) on the maximal energy dissipation. The intermediate regions were affected most as the maximal energy dissipation was significantly increased in IZC (91%), IZM (57%), and IZL (139%) (Figure 6a). Furthermore, regional variations were observed in the control group as the PB had a significantly higher maximal energy dissipation compared to the IZL. Following elastase treatment, maximal energy dissipation appeared to be the least in the AB, which was significantly different than IZC and IZM.

The effect of elastase treatment on the compressive instantaneous modulus is illustrated in Figure 6b. Two‐way ANOVA only revealed a significant effect of the disc region (*p* < .05) on the maximal energy dissipation. Following elastase treatment, there seemed to be a noticeable upward trend of instantaneous modulus in the thinnest regions (IZC, IZM, and IZL). In contrast, the thicker regions showed a decreasing response to the treatment, which was significantly pronounced in the AB (46%). The instantaneous modulus was regionally different in the control group as both AB and PB were significantly stiffer than IZC and IZL. The treaded group did not show any regional differences of instantaneous modulus.

### 3.7 TEM

**FIGURE 7:**
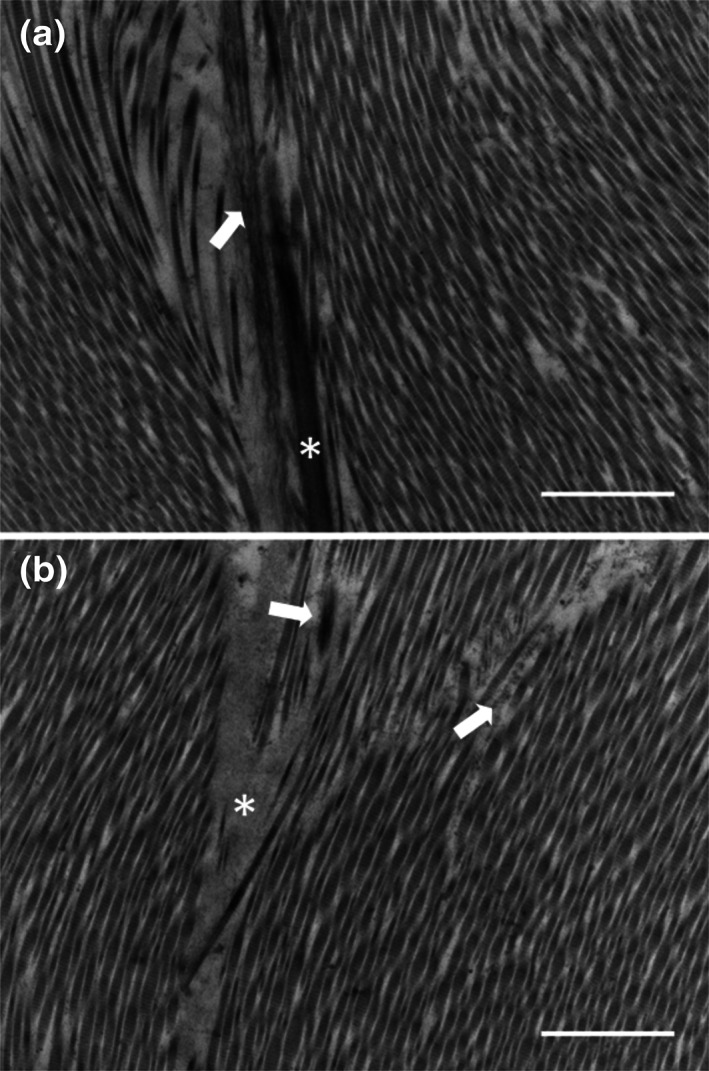
Transmission electron micrographs of IZC of porcine TMJ disc before (a) and after (b) elastase treatment. (a) Two different stages of elastin fiber development are visible: (1) the premature elastin fiber (white arrowhead) is seen as microfibrils, assembling together to create a layout where elastin will be deposited. Also, a mature elastin fiber (white asterisk) can be recognized by its amorphous elastin core and surrounding microfibrils. These fibers seem to be running along the collagen fibrils, which are distinguishable by their distinctive D‐periodic banding pattern. (b) Following elastase treatment, we could hardly spot any elastin fibers, except some elastin devoid areas in the middle of collagen fibrils (white asterisk), seemingly containing some remnants of microfibrils (white arrowhead) and elastin. Except the appearance of these void spaces, the collagen fibrils arrangement did not seem to be affected following the elastase treatment. Scale bar: 2 μm

A representative image of the IZC region of the control disc (Figure 7) demonstrates a highly ordered array of collagen fibrils. Microfibrils of elastin were observed both independently and in close association with amorphous elastin core, forming mature elastin fibers aligned parallel to the collagen fibrils (Figure 7a). Following elastase digestion, we could not identify any type of elastin fiber except some areas devoid of elastinin collagen interfibrillar spaces (Figure 7b). Furthermore, the collagen fibrils seemed to be less densely packed; nonetheless, the orientation of the fibrils was retained (Figure 7b).

## 4 DISCUSSION

Our study indicates a structural–functional role of elastin fibers in the disc. Immunofluorescent localization of elastin revealed a varying extent of elastin fragmentation especially in the intermediate zone after elastase treatment. Furthermore, we found a significant volume enlargement of the disc, reduction of collagen fibers tortuosity and cell shape elongation. Biochemical analysis confirmed the efficacy of the elastase treatment and its corresponding structural changes by showing significant reduction of elastin, collagen and GAG content. The resultant effect of these structural and compositional changes was reflected in the altered viscoelastic mechanical properties of the disc.

Following elastase treatment, the Mullins effect and nonlinear stiffening behavior of the control group still occurred in the corresponding stress–strain curves, meaning that the progressive engagement of collagen fibers were still taking place. Despite the degradation and reduction of elastin in different regions, the treated discs appeared to be elastic enough to retreat back to the zero‐strain state during the unloading cycle. However, the treated samples were experiencing continuous softening likely associated with inability of tissue to recover completely. This observation is in accordance with Schriefl et al. (2015) as they also observed continuous softening in elastase treated samples attributed to the continuous elongation of the tissue over the loading cycles.

In agreement with Yu, Tirlapur, Fairbank, and Handford (2007), we observed elastin fibers forming cross‐bridge connectivity between collagen fibers, especially in the AB. Probably, the collagen fibers are no longer anchored to the matrix or to one another at locations devoid of the elastin fibers, and hence they rearrange. The regions with significant loss of elastin fibers exhibited significant increase of maximal energy dissipation. Given the higher thickness in the AB and PB, our confocal images showed only partial degradation of elastin fibers in these regions, leaving more elastin fibers through the collagenous network; consequently, limited changes of their hysteresis properties were observed.

Elastase treatment also resulted in regionally different values of the instantaneous modulus. However, unlike the hysteresis data, two‐way ANOVA did not reveal an overall significant effect of elastase treatment on the instantaneous modulus. This may be due to the fact that its calculation is based on the maximal point of the stress–strain curve where the mechanical behavior of the tissue is primarily governed by collagen fiber. In accordance with previous studies (Chow et al., 2013; Fonck et al., 2007; Lee et al., 2001; Schriefl et al., 2015), we observed an increasing trend of the modulus in the intermediate zone of the treated discs, especially in the IZC and IZL. This stiffer behavior can be explained by a significant reduction of the collagen fibers tortuosity, yielding to collagen recruitment at lower strains. This phenomenon highlights a vital mechanical function of elastin fibers in the disc as they could provide shock absorption, taking up small strains and thereby shielding collagen fibers against repetitive impact loading (Chow et al., 2013; Fonck et al., 2007; Lee et al., 2001; Schriefl et al., 2015; Yapp & Chen, 2015).

With elastin being removed, the existing prestress on collagen fibers is abolished, leaving them in a relaxed state. Another study (Shah, Palacios, & Palacios, 1982) suggested a cell‐induced mechanism involving traction forces generated by fibrocartilage cells, which buckles the collagen fibers and create a periodic crimp structure. However, this view is opposed by Ghazanfari et al. (2015) who found formation of collagen crimps upon the appearance of elastin fibers in tissue engineered constructs. In light of this perspective, considering the collagen fibers acting as a contact guidance for the cells, having them straightened following the elastase digestion forces the cells elongate accordingly (Ghazanfari, Alberti, Xu, & Khademhosseini, 2019; Ghazanfari, Khademhosseini, & Smit, 2016; Ghazanfari, Werner, Ghazanfari, Weaver, & Smit, 2018). The significant elongation of cells after elastase treatment in our study might support this theory, but we cannot confirm it without further experiments.

The reduction of instantaneous modulus in the AB and PB is likely due to differences in orientation of collagen and elastin fibers, biochemical composition and thickness, which all impede the diffusion of elastase and compromise its degradative role. This is in line with a study of Chow et al. (2013) in which different mechanical behavior was attributed to the different structure of elastin lamella and uneven degradation of elastin fibers due to the thickness.

Interestingly, we found that elastase treatment caused significant reduction of collagen fibers and GAG content in some regions of the disc. Elastase has been described to facilitate collagen degradation by de‐masking collagen fibers through removal of the ground substance such as GAG and elastin, as well as depolymerizing the insoluble collagen (Ujiie, Shimada, Komatsu, & Gomi, 2008) or even directly denaturing the collagen fibers (Kafienah et al., 1998). By contrast, although collagenase is considered as the main collagenolytic enzyme, it cannot digest the highly crosslinked insoluble collagen polymers. This may explain the limited reduction of collagen content after the collagenase digestion of the disc in our previous study (Fazaeli et al., 2016) vis‐à‐vis the corresponding results we obtained after elastase digestion in this study.

Despite the presence of SBTI in our enzymatic solution, reduction of collagen content was not completely prevented. This finding is in agreement with other studies who reported different extent of this non‐specific degradation in presence (Smith et al., 2008) or absence (Barbir et al., 2010; Chow et al., 2013) of SBTI. Despite this inevitable side effect of elastase, we conclude that the mechanical changes in our study should not be attributed solely to the contribution of remaining collagenous network but to the fragmentation and reduction of elastin fibers. This is of significant importance as we observed a completely different structural and mechanical response to the collagenase digestion in our previous study (Fazaeli et al., 2016).

It is also interesting to note that while GAGs considerably contribute to the compressive mechanical properties of the articular cartilage, they seem to have a different role in the TMJ disc. This different contribution is attributed to the compositional and structural differences between the GAGs in the TMJ disc and articular cartilage. The significantly lower content, 20 times, of GAGs in the TMJ disc compared to that of the articular cartilage can be the main reason for a minor influence of GAGs to the mechanical properties of the TMJ disc. Willard, Kalpakci, Reimer, and Athanasiou (2012) showed that even 90% depletion of GAGs in the TMJ disc did not affect the instantaneous modulus of the treated discs. Interestingly, they observed a significant positive correlation between collagen content and compressive properties of the disc. Therefore, GAGs might play more of an indirect role in mechanical properties of the TMJ disc. As discussed in the presented study, elastin fibers likely compensate the minor restorative role of GAGs in the TMJ disc upon load removal. In articular cartilage however, GAGs are known to be a major contributor in maintaining homeostatic configuration of the cartilage.

In light of the presented findings, any structural and compositional changes of elastin fibers caused naturally through aging or pathologically as in degenerative conditions is of clinical importance. The elastin content is reported to decrease through advancing age and is not only accountable for the loss of elasticity of tissues but also provokes structural changes of collagenous network, potentially leading to irreversible mechanical changes in the tissue (Yu et al., 2007). Regarding the diarthrodial joint, a higher level of elastase has been reported in the synovial fluid of rheumatoid arthritis (Huet, Flipo, Richet, & Thiebaut, 1992; Virca, Mallya, Pepys, & Schnebli, 1984). As in case of the TMJ, different components are exposed to the synovial fluid, which could be affected by presence of proteases in it (Hall, Brown, & Baughman, 1984). We show here that degradation of elastin fibers influences its viscoelastic properties; this could subject it to unfavorable biomechanics during the jaw movement; thereby affecting kinematics of the disc. This may lead to malpositioning of the disc relative to the mandibular and condylar bone, known as internal derangement (Senga, Mizutani, Kobayashi, & Ueda, 1999).

The present results point out that the contribution of elastin fibers to the TMJ disc biomechanics goes beyond merely a recovering role; influencing on the viscoelastic properties of the disc through an interaction, mainly with collagen fibers and other ECM components. Given the age‐dependent alteration of biochemical composition and structural–functional characteristics of the TMJ disc, the findings should be interpreted cautiously for aged porcines. It is hoped that this study offers a better insight on the potential degradative contribution of elastase in pathophysiological conditions and also provides a stepping stone toward development of new and improved tissue engineering strategies by bringing elastin fibers into the perspective.

## Supporting information


**Figure S1**. Image analysis procedure to calculate the shape index. (a) A representative DAPI image used for quantification of the cell shape, and (b) the resulting binary image after the geometrical filter was applied.

**Figure S2**. Representative confocal imaging and immunofluorescence staining of elastin fibers, collagen fibers type I and cell nuclei from the superior surface of IZC in the porcine TMJ disc before and after elastase treatment. Elastin fibers, collagen fibers type I, and cell nuclei can be distinguished in red, green, and blue respectively. The schematic configuration of the TMJ disc sagittal cross‐section, placed in the uppermost center of the figure exhibits the superior surface of IZC region in the TMJ disc. The upper row images show the overlay and separate immunofluorescence staining of the control (PBS) samples and the lower row shows the treated (elastase) ones. The overlay immunofluorescence staining of the control (PBS) sample exhibits that collagen fibers run anterioposteriorly at the superior surface where elastin fibers and cell nucleuses are present in abundance. In the treated (elastase) sample however, the collagen fibers seemed to be more disorganized near the surface where elastin fibers were severely disintegrated and cell nucleuses were markedly diminished. Scale bar: 50 μm.

**Figure S3**. Top view confocal imaging and immunofluorescence staining of elastin fibers of porcine TMJ disc before and after elastase treatment. The schematic configuration of the TMJ disc (seen from the top), placed in the uppermost right corner of the figure exhibits the location of different regions of the TMJ disc and the direction of imaging (top view). Regions labeled with asterisk present the treated (elastase) samples. Note the frequent oblique elastin fibers criss‐crossing through the isotropic collagenous network at the AB, while in other regions, elastin fibers are mainly aligned parallel with collagen fibers. Following the elastase treatment (regions with asterisk), the elastin fibers are fragmented, patched and diminished across the TMJ disc. Scale bar: 50 μm.

**Figure S4**. Top view confocal imaging and immunofluorescence staining of collagen fibers type I of porcine TMJ disc before and after elastase treatment. The schematic configuration of the TMJ disc (seen from the top), placed in the uppermost right corner of the figure exhibits the location of different regions of the TMJ disc and the direction of imaging (top view). Regions labeled with asterisk present the treated (elastase) samples. Anteroposteriorly aligned collagen fibers in the intermediate regions (IZC, IZM, and IZL) merge with fibers at the peripheral bands (PB and AB), forming a dense collagenous network. Note the reduction of collagen fibers tortuosity following the elastase treatment (regions with asterisk), with more noticeable impact in the intermediate regions (IZC, IZM, and IZL). Scale bar: 50 μm.

**Figure S5**. Top view confocal imaging and immunofluorescence staining of cell nuclei of porcine TMJ disc before and after elastase treatment. The schematic configuration of the TMJ disc (seen from the top), placed in the uppermost right corner of the figure exhibits the location of different regions of the TMJ disc and the direction of imaging (top view). Regions labeled with asterisk present the treated (elastase) samples. Note the cell nucleuses elongation following the elastase treatment (regions with asterisk), with more noticeable effect in the intermediate regions (IZC, IZM, and IZL). Scale bar: 50 μm.

**Figure S6**. Sagittal view confocal imaging and immunofluorescence staining of collagen fibers type I of porcine TMJ disc before and after elastase treatment. The schematic configuration of the TMJ disc sagittal cross‐section, placed in the uppermost right corner of the figure exhibits the location of different regions of the TMJ disc. The upper row shows images of different regions in the control (PBS) samples and the lower row shows the treated (elastase) ones. The collagen fibers, running anteroposteriorly in the IZC, extend to the AB and PB where they merged with fibers mainly aligned mediolaterally (perpendicular to the plane). Note the reduction of collagen fibers tortuosity, with more noticeable impact in the intermediate regions (IZC, IZM, and IZL). Scale bar: 50 μm.

**Figure S7**. Sagittal view confocal imaging and immunofluorescence staining of collagen fibers type I of porcine TMJ disc before and after elastase treatment. The schematic configuration of the TMJ disc sagittal cross‐section, placed in the uppermost right corner of the figure exhibits the location of different regions of the TMJ disc. The upper row shows images of different regions in the control (PBS) samples and the lower row shows the treated (elastase) ones. The collagen fibers, running anteroposteriorly in the IZC, extend to the AB and PB where they merged with fibers mainly aligned mediolaterally (perpendicular to the plane). Note the reduction of collagen fibers tortuosity, with more noticeable impact in the intermediate regions (IZC, IZM, and IZL). Scale bar: 50 μm.

**Figure S8**. Sagittal view confocal imaging and immunofluorescence staining of cell nuclei of porcine TMJ disc before and after elastase treatment. The schematic configuration of the TMJ disc sagittal cross‐section, placed in the uppermost right corner of the figure exhibits the location of different regions of the TMJ disc. The upper row shows images of different regions in the control (PBS) samples and the lower row shows the treated (elastase) ones. Note the diminishing and elongation of cell nucleuses, with more noticeable impact in the intermediate regions (IZC, IZM, and IZL). Scale bar: 50 μm.

**Figure S9**. Representative confocal imaging and immunofluorescence staining of elastin fibers, collagen fibers type I and cell nuclei of porcine artery. Elastin fibers, collagen fibers type I and cell nuclei can be distinguished in red, green, and blue respectively. On the right is lumen (L) of the artery and on the left, is the media (M). In the control (PBS). Scale bar: 50 μm.
