# Supplementary figures and images for: Alteration of structural and mechanical properties of the temporomandibular joint disc following elastase digestion
Source: J Biomed Mater Res B Appl Biomater. 2020 Jun 1;108(8):3228–40. doi: 10.1002/jbm.b.34660 (PMC7586824; doi:10.1002/jbm.b.34660)

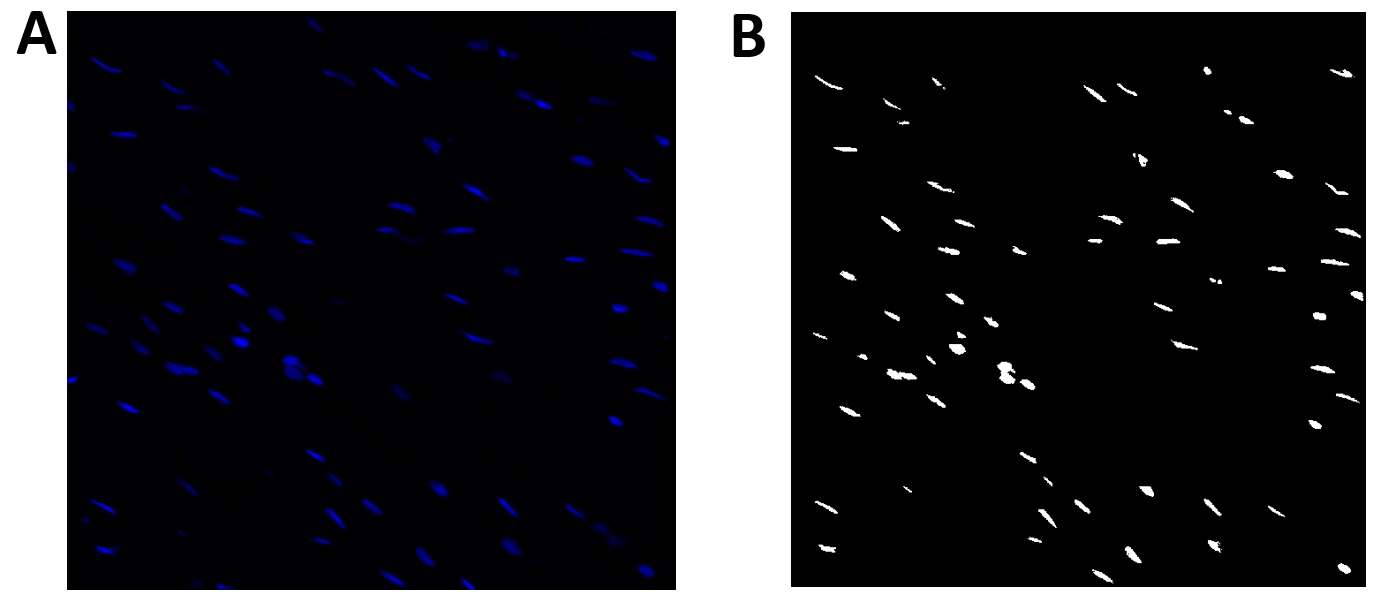

Supplement: Supplementary file 1 — Figure S1. Image analysis procedure to calculate the shape index. (a) A representative DAPI image used for quantification of the cell shape, and (b) the resulting binary image after the geometrical filter was applied. [file JBM-108-3228-s001.tif]

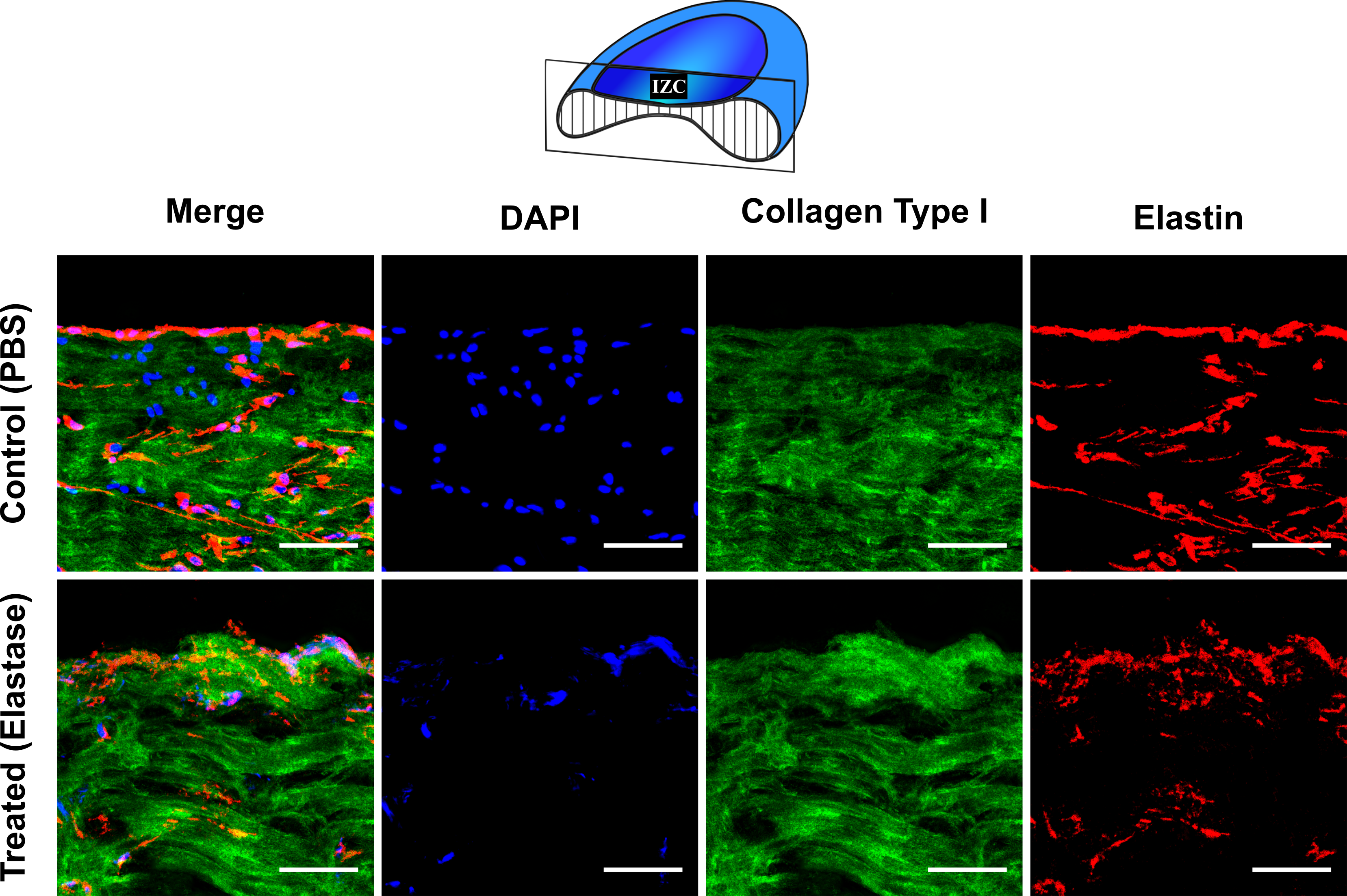

Supplement: Supplementary file 2 — Figure S2. Representative confocal imaging and immunofluorescence staining of elastin fibers, collagen fibers type I and cell nuclei from the superior surface of IZC in the porcine TMJ disc before and after elastase treatment. Elastin fibers, collagen fibers type I, and cell nuclei can be distinguished in red, green, and blue respectively. The schematic configuration of the TMJ disc sagittal cross‐section, placed in the uppermost center of the figure exhibits the superior surface of IZC region in the TMJ disc. The upper row images show the overlay and separate immunofluorescence staining of the control (PBS) samples and the lower row shows the treated (elastase) ones. The overlay immunofluorescence staining of the control (PBS) sample exhibits that collagen fibers run anterioposteriorly at the superior surface where elastin fibers and cell nucleuses are present in abundance. In the treated (elastase) sample however, the collagen fibers seemed to be more disorganized near the surface where elastin fibers were severely disintegrated and cell nucleuses were markedly diminished. Scale bar: 50 μm. [file JBM-108-3228-s002.tif]

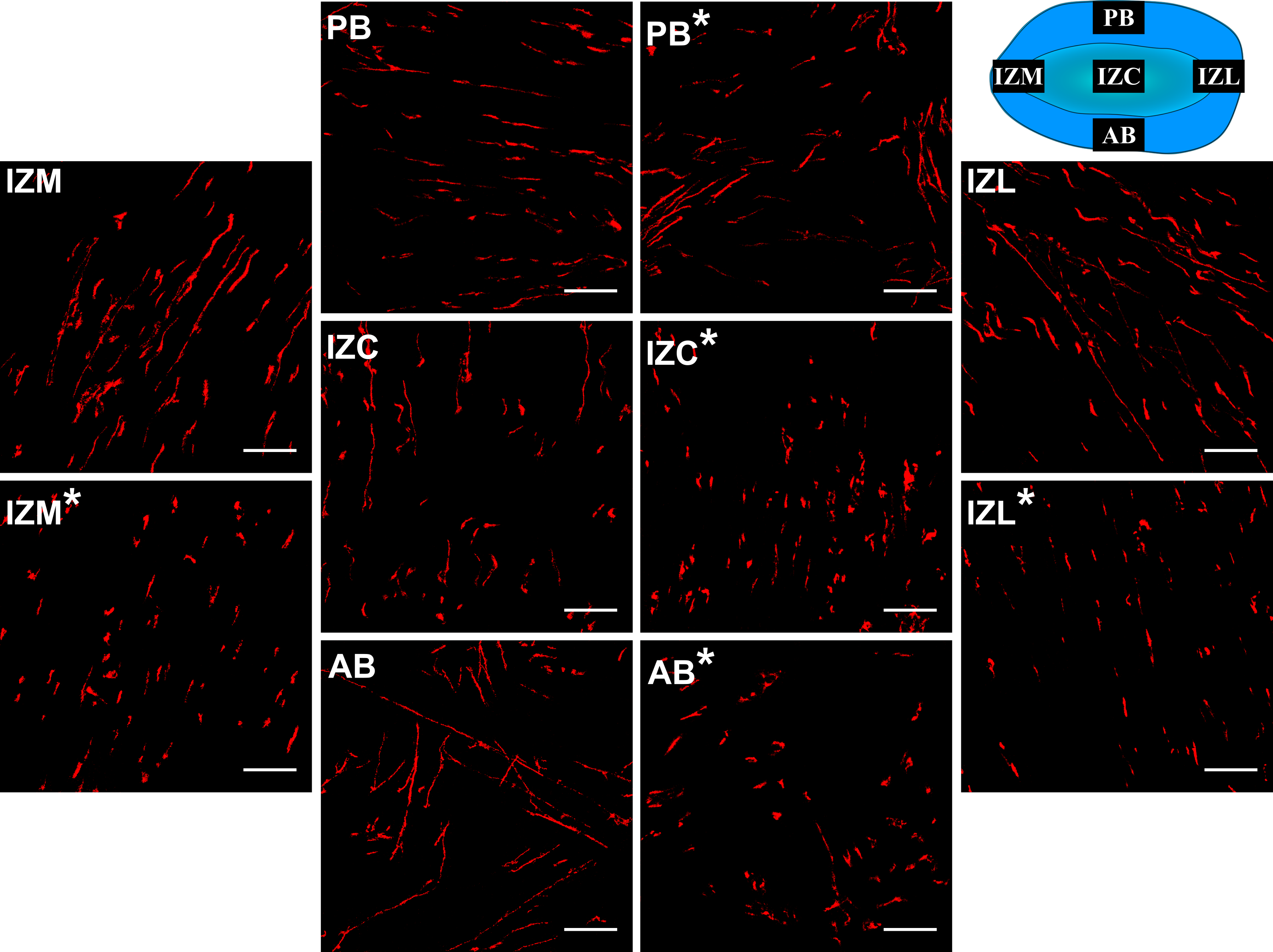

Supplement: Supplementary file 3 — Figure S3. Top view confocal imaging and immunofluorescence staining of elastin fibers of porcine TMJ disc before and after elastase treatment. The schematic configuration of the TMJ disc (seen from the top), placed in the uppermost right corner of the figure exhibits the location of different regions of the TMJ disc and the direction of imaging (top view). Regions labeled with asterisk present the treated (elastase) samples. Note the frequent oblique elastin fibers criss‐crossing through the isotropic collagenous network at the AB, while in other regions, elastin fibers are mainly aligned parallel with collagen fibers. Following the elastase treatment (regions with asterisk), the elastin fibers are fragmented, patched and diminished across the TMJ disc. Scale bar: 50 μm. [file JBM-108-3228-s003.tif]

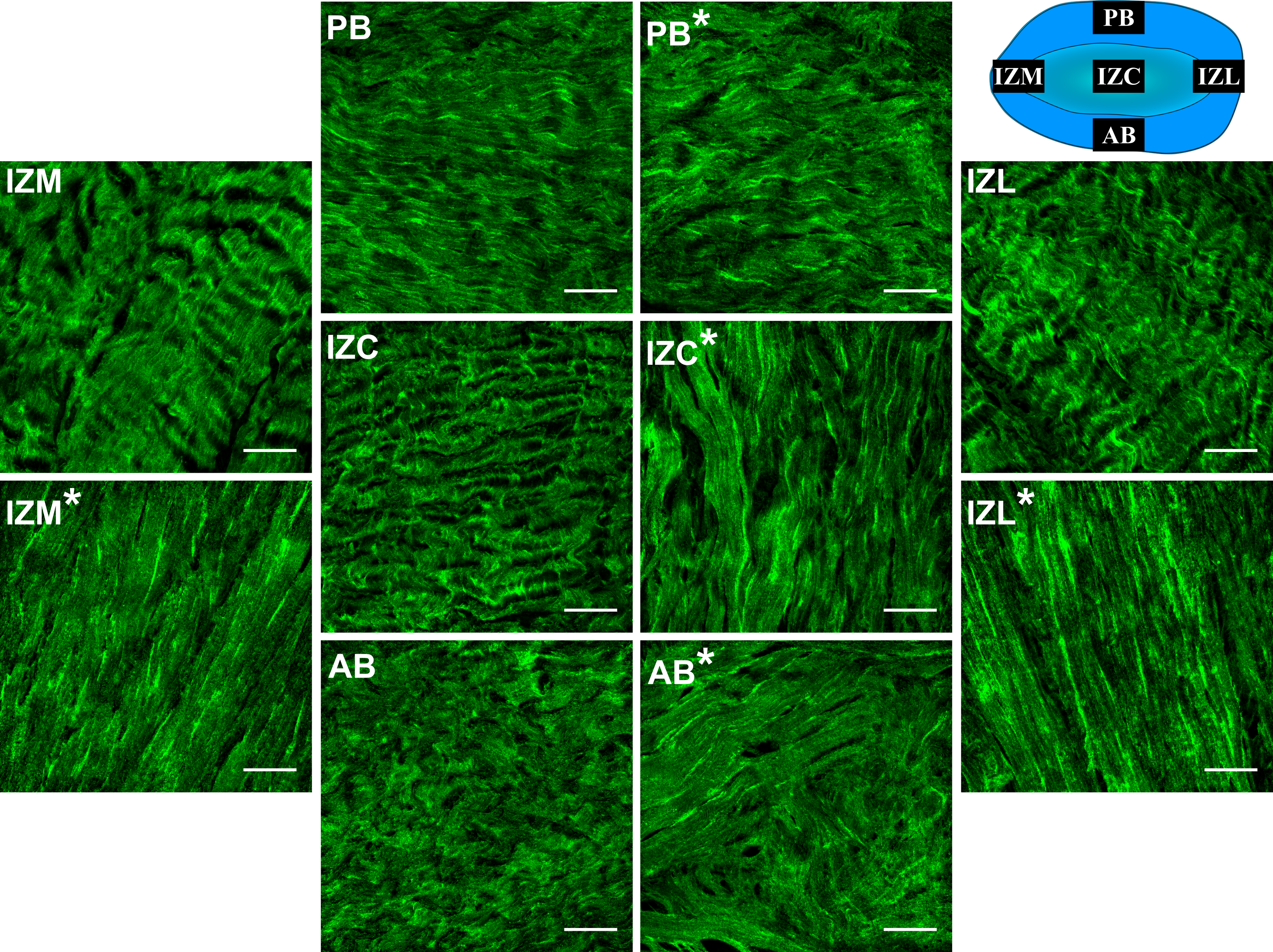

Supplement: Supplementary file 4 — Figure S4. Top view confocal imaging and immunofluorescence staining of collagen fibers type I of porcine TMJ disc before and after elastase treatment. The schematic configuration of the TMJ disc (seen from the top), placed in the uppermost right corner of the figure exhibits the location of different regions of the TMJ disc and the direction of imaging (top view). Regions labeled with asterisk present the treated (elastase) samples. Anteroposteriorly aligned collagen fibers in the intermediate regions (IZC, IZM, and IZL) merge with fibers at the peripheral bands (PB and AB), forming a dense collagenous network. Note the reduction of collagen fibers tortuosity following the elastase treatment (regions with asterisk), with more noticeable impact in the intermediate regions (IZC, IZM, and IZL). Scale bar: 50 μm. [file JBM-108-3228-s004.tif]

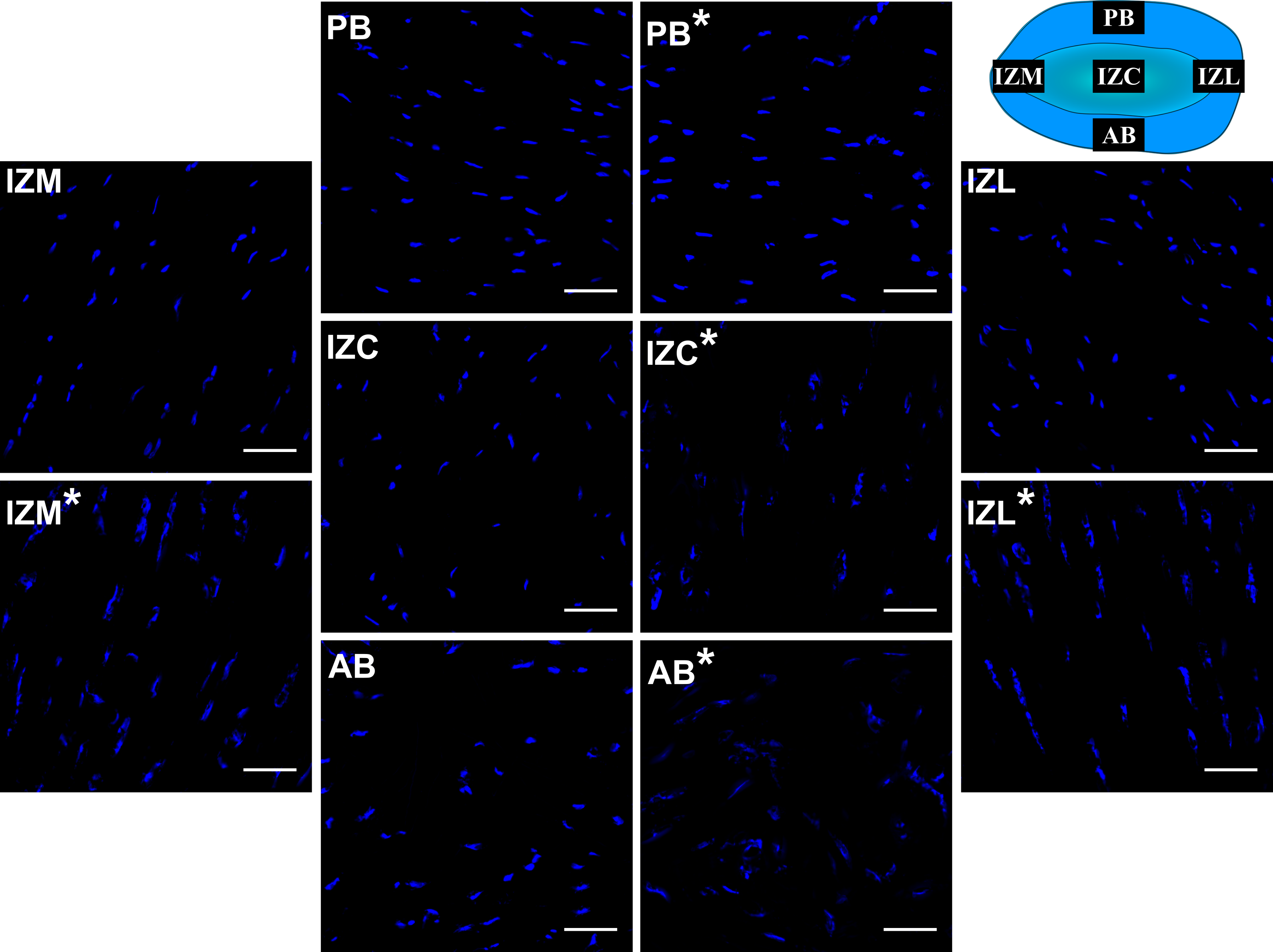

Supplement: Supplementary file 5 — Figure S5. Top view confocal imaging and immunofluorescence staining of cell nuclei of porcine TMJ disc before and after elastase treatment. The schematic configuration of the TMJ disc (seen from the top), placed in the uppermost right corner of the figure exhibits the location of different regions of the TMJ disc and the direction of imaging (top view). Regions labeled with asterisk present the treated (elastase) samples. Note the cell nucleuses elongation following the elastase treatment (regions with asterisk), with more noticeable effect in the intermediate regions (IZC, IZM, and IZL). Scale bar: 50 μm. [file JBM-108-3228-s005.tif]

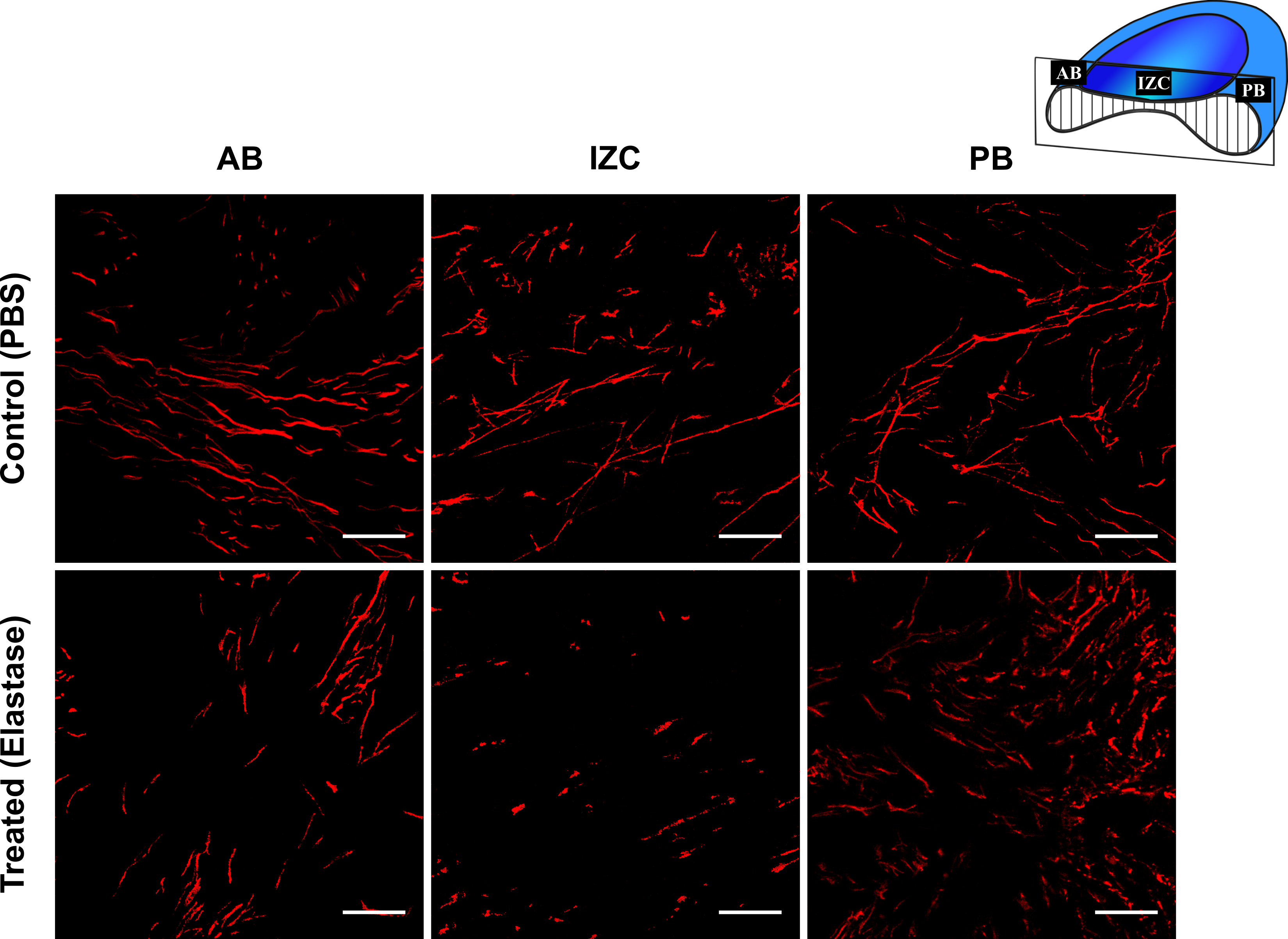

Supplement: Supplementary file 6 — Figure S6. Sagittal view confocal imaging and immunofluorescence staining of collagen fibers type I of porcine TMJ disc before and after elastase treatment. The schematic configuration of the TMJ disc sagittal cross‐section, placed in the uppermost right corner of the figure exhibits the location of different regions of the TMJ disc. The upper row shows images of different regions in the control (PBS) samples and the lower row shows the treated (elastase) ones. The collagen fibers, running anteroposteriorly in the IZC, extend to the AB and PB where they merged with fibers mainly aligned mediolaterally (perpendicular to the plane). Note the reduction of collagen fibers tortuosity, with more noticeable impact in the intermediate regions (IZC, IZM, and IZL). Scale bar: 50 μm. [file JBM-108-3228-s006.tif]

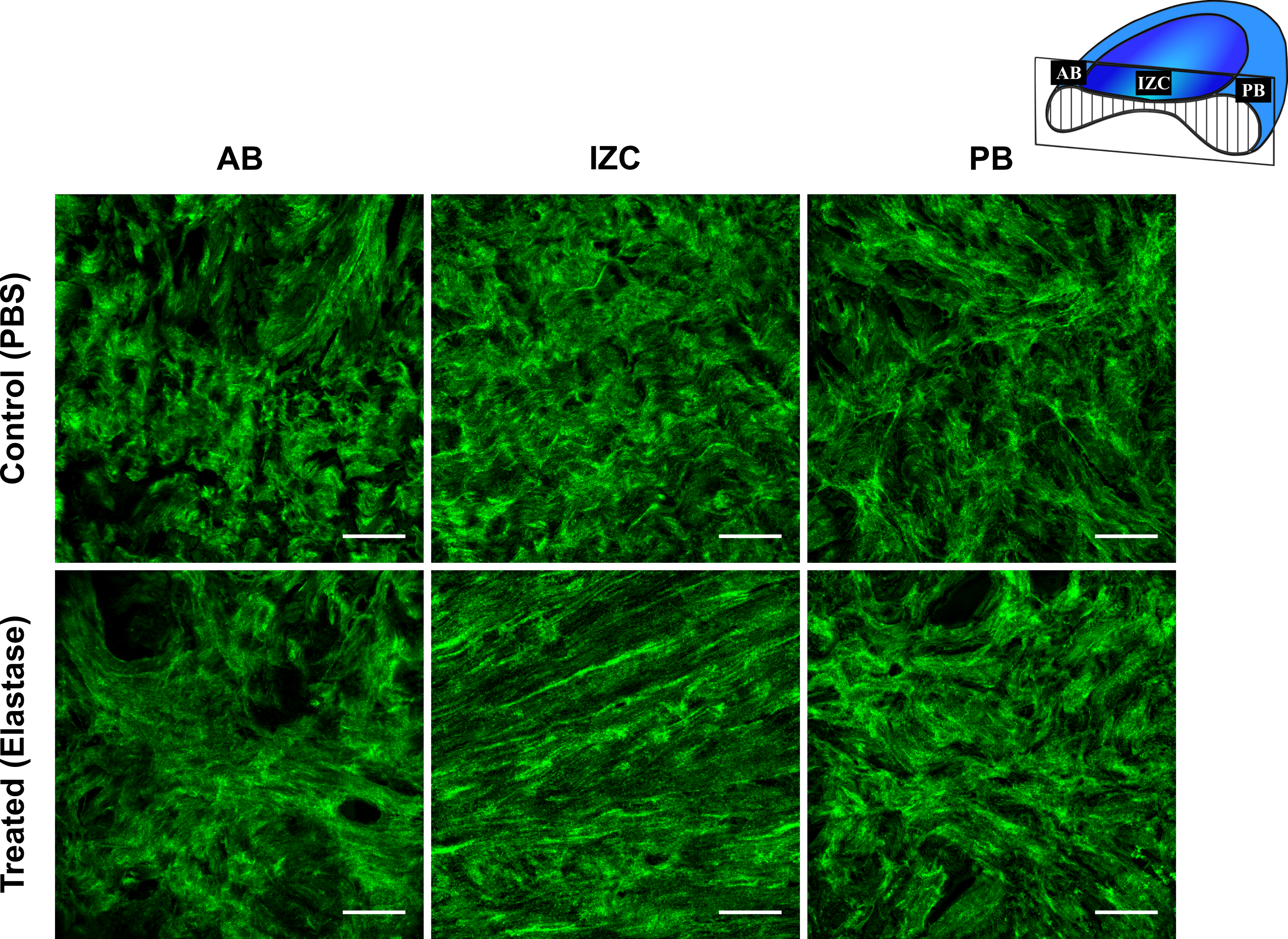

Supplement: Supplementary file 7 — Figure S7. Sagittal view confocal imaging and immunofluorescence staining of collagen fibers type I of porcine TMJ disc before and after elastase treatment. The schematic configuration of the TMJ disc sagittal cross‐section, placed in the uppermost right corner of the figure exhibits the location of different regions of the TMJ disc. The upper row shows images of different regions in the control (PBS) samples and the lower row shows the treated (elastase) ones. The collagen fibers, running anteroposteriorly in the IZC, extend to the AB and PB where they merged with fibers mainly aligned mediolaterally (perpendicular to the plane). Note the reduction of collagen fibers tortuosity, with more noticeable impact in the intermediate regions (IZC, IZM, and IZL). Scale bar: 50 μm. [file JBM-108-3228-s007.tif]

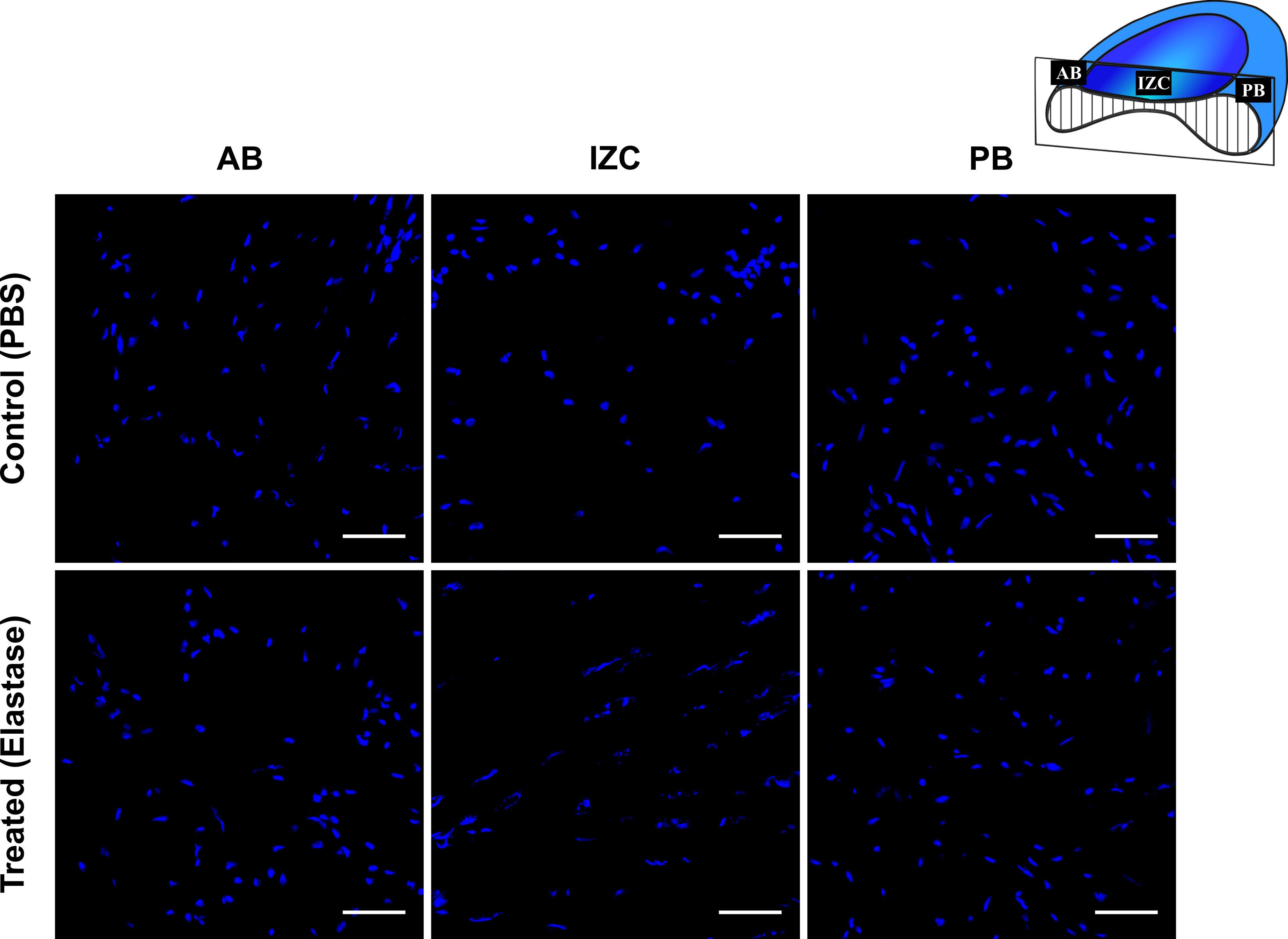

Supplement: Supplementary file 8 — Figure S8. Sagittal view confocal imaging and immunofluorescence staining of cell nuclei of porcine TMJ disc before and after elastase treatment. The schematic configuration of the TMJ disc sagittal cross‐section, placed in the uppermost right corner of the figure exhibits the location of different regions of the TMJ disc. The upper row shows images of different regions in the control (PBS) samples and the lower row shows the treated (elastase) ones. Note the diminishing and elongation of cell nucleuses, with more noticeable impact in the intermediate regions (IZC, IZM, and IZL). Scale bar: 50 μm. [file JBM-108-3228-s008.tif]

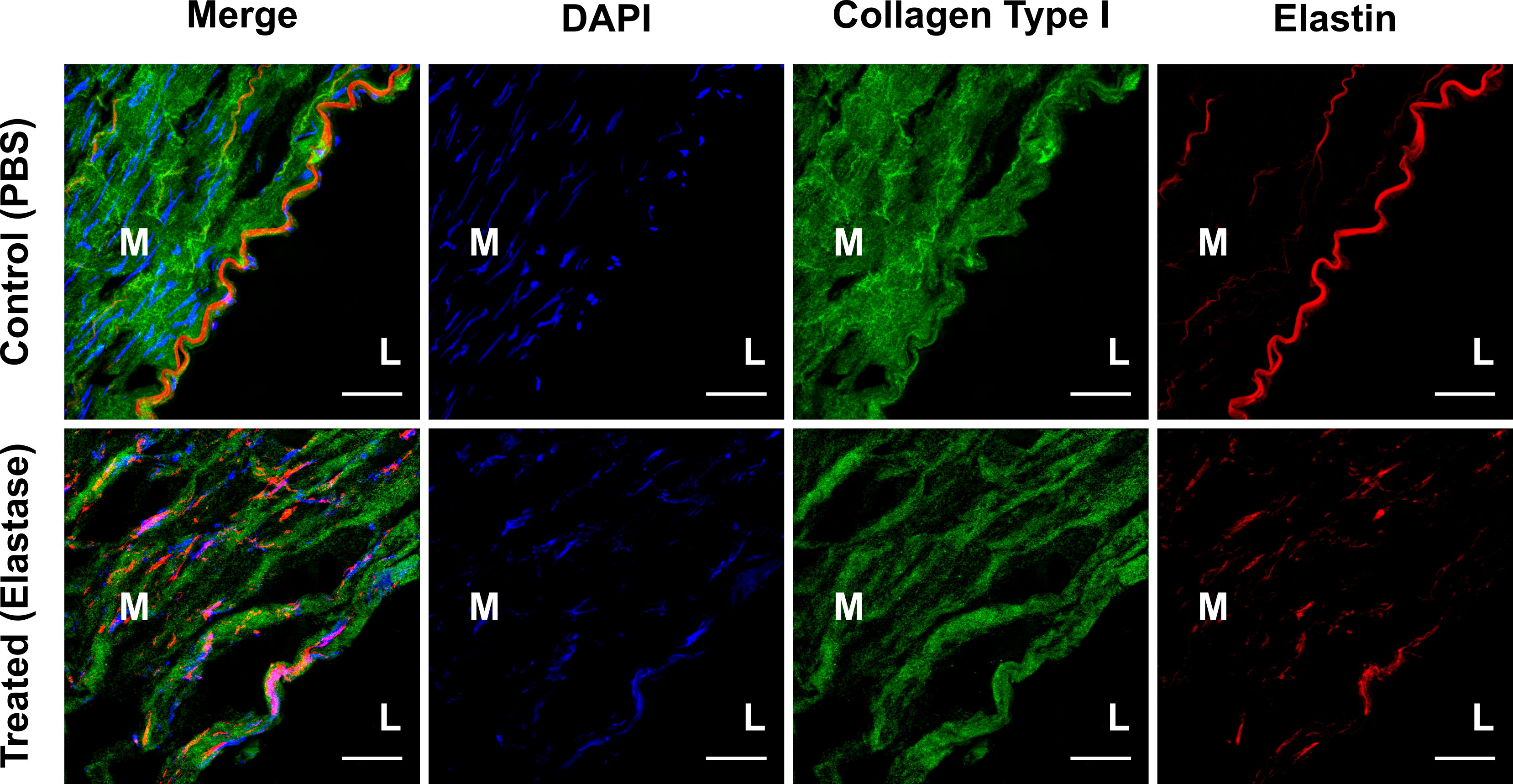

Supplement: Supplementary file 9 — Figure S9. Representative confocal imaging and immunofluorescence staining of elastin fibers, collagen fibers type I and cell nuclei of porcine artery. Elastin fibers, collagen fibers type I and cell nuclei can be distinguished in red, green, and blue respectively. On the right is lumen (L) of the artery and on the left, is the media (M). In the control (PBS). Scale bar: 50 μm. [file JBM-108-3228-s009.tif]
